# CD8^+^ T Lymphocytes: Crucial Players in Sjögren’s Syndrome

**DOI:** 10.3389/fimmu.2020.602823

**Published:** 2021-01-28

**Authors:** Huimin Zhou, Jun Yang, Jie Tian, Shengjun Wang

**Affiliations:** ^1^ Department of Laboratory Medicine, The Affiliated People’s Hospital, Jiangsu University, Zhenjiang, China; ^2^ Department of Immunology, Jiangsu Key Laboratory of Laboratory Medicine, School of Medicine, Jiangsu University, Zhenjiang, China

**Keywords:** Sjögren’s syndrome, CD8^+^ T lymphocyte, pathophysiology, tissue resident lymphocyte, immune regulation

## Abstract

Primary Sjögren’s syndrome (pSS) is a chronic autoimmune disease associated with damage to multiple organs and glands. The most common clinical manifestations are dry eyes, dry mouth, and enlarged salivary glands. Currently, CD4^+^ T lymphocytes are considered to be key factors in the immunopathogenesis of pSS, but various studies have shown that CD8^+^ T lymphocytes contribute to acinar injury in the exocrine glands. Therefore, in this review, we discussed the classification and features of CD8^+^ T lymphocytes, specifically describing the role of CD8^+^ T lymphocytes in disease pathophysiology. Furthermore, we presented treatment strategies targeting CD8^+^ T cells to capitalize on the pathogenic and regulatory potential of CD8^+^ T lymphocytes in SS to provide promising new strategies for this inflammatory disease.

## Introduction

Sjögren’s syndrome (SS) is defined as primary SS (pSS) or secondary SS (sSS) which is associated with other autoimmune diseases (usually rheumatoid arthritis (RA), lupus, or scleroderma). pSS may occur alone and is a chronic autoimmune disease characterized by dry mouth and eyes. pSS patients often have distinct clinical symptoms, such as slow salivary flow rates and decreased salivary mucin quality, accompanied by dry keratitis or conjunctivitis (1, 2). Glandular lesions in patients with pSS are characterized by the mass infiltration of inflammatory cells and formation of ectopic germinal centers (GCs), which are anatomically and functionally similar to the GCs found in the secondary lymphatic organs (3). These inflammatory cells include T lymphocytes, B lymphocytes, natural killer cells (NKs), dendritic cells (DCs) and macrophages (2, 4).

CD8^+^ T lymphocytes are a complex group of lymphocytes with different phenotypes which are known to display a critical role in tumor, viral infection, chronic inflammation, and autoimmune disease (5–8). Overactivity or abnormal proliferation of CD8^+^ T lymphocytes can be detected in the peripheral circulation and specific target tissues of SS patients (9, 10). In the damaged lacrimal and salivary glands of SS patients or non-obese diabetic (NOD) murine models, there are always some CD8^+^ T cells among the infiltrating T cells, even though the number of CD4^+^ T cells is higher. It has been observed that activated CD8^+^ T lymphocytes accumulate around apoptotic acinar epithelial cells, and the ascensive effector molecules of cytotoxic CD8^+^ T lymphocytes (CTLs) such as Granzyme B (GrB), perforin, interferon-*γ* (IFN-*γ*), and tumor necrosis factor-α (TNF-α) can be detected (11, 12). These results indicate the presence of a population of activated CD8^+^ T lymphocytes with cytotoxicity that are capable of killing the glandular epithelial cells and leading to the death or apoptosis of the cells. However, some researchers have found that CD8^+^ T cells located in the ocular surface of a murine model could regulate Th17 cells and prevent disease progression (13). Therefore, the pathogenicity and regulatory effects of CD8^+^ T cells still require further clarification.

The distinctive role of CD8^+^ T lymphocytes in SS is likely influenced by effector molecules and cellular distribution. Here, we discuss our current understanding of the involvement of CD8^+^ T lymphocytes in lacrimal and salivary gland injury during SS development and review possible targeted therapies for CD8^+^ T lymphocytes.

## CD8^+^ T Lymphocytes as Effector T Cells: Pathogenicity

Traditionally, naïve CD8^+^ T lymphocytes expressing the surface markers CD27, CCR7, and CD45RA are located in the secondary T cell zone of the thymus gland. When professional antigen presenting cells (APCs), such as DCs and B cells, present specific antigens on major histocompatibility complex 1 (MHC-1) molecules to CD8^+^ T cells together with co-stimulatory molecules to release cytokine signaling, CD8^+^T cells are activated, express CD57, and become cytotoxic T lymphocytes (CTLs) (14). As previously described, there was a negative correlation between perforin and CD27 expression, and CD27^low/−^CD8^+^ T cell subsets showed high cytolytic activity, indicating that these subsets were mainly cytotoxic effector T cells. Thus, mature CTLs are CD27^−^CD28^–^CD45RA^+^ T cells (15).

### Classification, Features, and Functions

Effector CD8^+^ T lymphocytes play a vital role in killing tumor cells and controlling pathogen-infected cells. CTLs directly affect target cells mainly through the release of GrB and perforin to clear target cells directly or Fas ligands (FasL), which is induced to bind to Fas death receptors on target cells, triggering apoptosis signalling (16). In addition to directly targeting infected cells, CTLs are likely to affect the occurrence and prognosis of diseases indirectly through the release of effector cytokines. This subset of CTLs expresses lower levels of GrB and perforin than other subsets but is more likely to participate in adaptive immune responses through a variety of proinflammatory cytokines, such as IFN-*γ*, IL-4, IL-17 and TNF-α. At present, it is believed that effector CD8^+^ T lymphocytes can be mainly divided into Tc1, Tc2, Tc9, Tc17, follicular cytotoxic T (Tfc), follicular helper T (CD8^+^ Tfh) and regulatory T (CD8^+^ Treg) cells in response to different stimuli, such as tumors, viral infections, allergies, autoimmune diseases and transplantation (17, 18) (**Table 1**).

**Table 1:** Diverse subsets of CD8^+^T lymphocytes and their respective effects.

CD8^+^ T Cell Subsets	Production	Mechanism	Effects	Location	Refs
CXCR5^+^ PD-1^int-^CD8^+^ Tfc cells (Bcl6^low^ TCF-1^low^)	IFN-*γ*; IL-21; Perforin; GrB	Little impact on GC without affecting follicular B cells; Cytotoxicity similar to that of CXCR5^−^CD8^+^ T cells and follicular B cells with viral infection;	Strong reaction to blockade by a PD-1 inhibitor The only killer cell associated with chronic viral infection Cancer treatment;	Blood of patients infected with LCMV, HIV, and HBV	(19, 20)
CXCR5^+^ PD-1^high^CD28^+^ CD40L^+^CD8^+^Tfh cells	IFN-*γ*; IL-21 IL-33; Costimulatory molecule iCOS, Batf, Maf, Nfatc, Irf4; Inhibitory receptors (Lag3, Pdcd1, Ctla4)	Up-regulation of CD70, OX40, and iCOS after activation to interact with B cells	Synergistically or independently interact with CD4^+^ T cells Assist in B activation; Maintain GCs; Promote autoantibody production and autoimmune diseases	RA synovial ectopic follicles; human tonsil and sinus follicular B cells	(19, 21–24)
CXCR5^+^Foxp3^+/−^CD8^+^Treg cells (CD44^+^CD122^+^Ly49^+^)	TCR; NKG2A	In combination with Qa-1 molecules	Inhibit DCs activation; reduce CD4^+^Tfh and GCB cells	Ocular surface of murine model of SS; blood of murine model of SLE, EAE or humans	(13, 25–32)
CXCR5^+^ CD8^+^ T_ab-supp_ cells	IFN-*γ*	Inhibits IL-4 expression by allo-specific CD4^+^ T cells	Directly kill allo-primed IgG^+^ B cells; Inhibit CD4^+^ Tfh and GCB cells to improve survival rate after transplantation		(28)
CXCR5^-^CXCR3^+^CD8^+^ Tc1 cells	Abundant soluble molecules such as IFN-γ, TNF-α, Perforin, GrB;	High cytotoxicity; Fas-FasL death pathway	Eliminate primary infection and malignant cells Anti-viral and antitumor effects; kill target cells directly or induce apoptosis	Surround apoptotic acinar epithelial cells of the lacrimal gland in SS patients; in the lacrimal and salivary glands of SS murine model such as NOD/Lgals1^−/−^ mice; IDDM	(20, 33–39)
CXCR5^−^ CD8^+^Tc2 cells	IL-4; IL-5		Deregulation of CTL function; Participate in specific mutagenesis	RA;	(40–42)
CXCR5^−^ CD8^+^ Tc3 cells;	IL-17		Loss of dissolution	CSF of MS, EAE;	(40, 43)
Classic memory CD44^hi^ CD8^+^ T cells	IL-7 receptor	Mediated by TCR, IL-17, IL-15 without infection; CD28, TLR2, IL-12 activation during infection	Differentiate into effector T cells quickly; Express IFN-*γ*, TNF-α, Perforin, GrB, CCL3, CCL4 and CCL5	T_CM_: secondary lymphoid organs; T_EM_: peripheral lymphoid tissues and blood	(44–52)
CD69^+^CD103^+^ CD49a^+^CD8^+^ T_RM_ cells	IFN-*γ*; GrB; Perforin	IFN-*γ* recruits the chemokines CXCL9 and CXCL10;CD103 binds to E-cadherin on epithelial cells	Migration to submandibular glands and exert effects in tissues; colocalize with acinar and ductal epithelial cells and destroy glandular tissue	Submandibular glands of p40^−/−^CD25^−/−^ murine model of SS; Peripheral non-lymphoid tissues during infection or vaccination	(53–57)
Naïve CD8^+^ T cells	CDRA, CCR7	Stimulated by IL-6 or IL-23, TGF-β	Differentiate into Tfc or Tfh cells		(58)

According to the main effector factors and phenotype, CXCR5^−^CTLs are mostly known as Tc1 cells and kill target cells in the context of antitumor immunity. These cells secrete IFN-*γ* and TNF-α and have high cytotoxicity (40). Tc1 cells are pathogenic in insulin-dependent diabetes mellitus (IDDM) but protective in allergic airway inflammation (33). Compared with Tc1 cells, Tc2 cells have lower cytotoxic functions and produce IL-4 and IL-5 in response to specific allergens (33, 40). Tc2 cells can produce IL-13 to enhance inflammatory responses, but these cells are less cytotoxic in diabetes than Tc1 cells (41). In addition, Tc2 cells may be involved in the development of RA by selective enrichment of IL-4-producing CD8^+^ T cells (42). Tc9 cells promote airway inflammation through Th2 cells while inhibiting CD4^+^T cell-mediated colitis (59). Tc17 cells (also known as Tc3 cells) mainly secrete IL-17 without cytolysis (40). Tc17 cells are present in the cerebrospinal fluid (CSF) of patients with multiple sclerosis (MS) and are crucial for Th17-mediated experimental autoimmune encephalomyelitis (EAE) and Th1-mediated autoimmune diabetes (43) (**Table 1**). However, these CTL subtypes have not been determined in SS yet and need further exploration.

CXCR5^+^CTLs are characterized by the transcription factor Bcl6 and can be further divided into three subgroups. First, Tfc cells are the only subgroup with cytotoxic abilities in chronic viral infections. These cells are also known as the progenitors of exhausted T cells (T_EX_ cells) and show higher proliferation and cytotoxicity than CXCR5^-^CTLs (40, 60). Tfc cells are particularly responsive to inhibitor checkpoint blockade, especially anti-PD-1 therapy and are excellent for treating cancer and chronic viral infections (19, 20, 34). Second, CD8^+^ Tfh cells interact with B cells and are recruited through CXCL13, producing high levels of IFN-*γ* and IL-21 and promoting autoimmune responses (21–24, 61, 62). The third subgroup is CD8^+^ Treg cells. QA-1-restricted CD8^+^ Treg cells inhibit Tfh- and Th17-mediated responses in diseases such as lupus-like syndrome, EAE and arthritis (25, 63, 64). Due to their special functions, Treg cells will be described in detail in the following section.

### Distribution of CTLs in Peripheral Blood

The marked activation of T lymphocytes in the peripheral blood of patients with Sjögren’s syndrome shows increased HLA-DR expression. Yukinobu et al. showed that the percentage of peripheral HLA-DR^+^CD11^−^CTLs but not HLA-DR^+^CD11^+^CD8^+^T (T-suppressed) cells in SS patients was obviously high compared with that of the control (9). In addition, serum immunoglobulin levels were positively correlated with the percentage of HLA-DR^+^CTLs and T-suppressed cells (9). Surprisingly, only CD8^+^ T cells but not CD4^+^ T cells were associated with HLA-DR up-regulation in damaged glandular tissue (10). Moreover, the expression of HLA-DR in glandular CD8^+^ T lymphocytes was closely related to the clinical symptoms of SS, including the European League Against Rheumatism Sjögren’s syndrome Disease Activity Index (ESSDAI) scores, suggesting that effector CD8^+^ T lymphocytes have a pathogenic role (9, 15). The pathogenic factors of SS are not clear, but it is thought that viruses are important inducers. In general, CD8^+^ T cells play a key role in the clearance of viral infections. In addition, it showed that dysregulated SS gene signatures (SGS), including epigenomes, mRNAs and proteins in peripheral blood of SS patients, significantly overlapped with SS-causing genes which mainly involved in interferon signal and a disintegrin and metalloproteinase (ADAM) substrates (65). Most significantly, the SS pathogenic genes were specifically associated with activated CTLs, which may reflect disease characteristics. Remarkably, transcriptional module 1 (TR1) in CD8^+^T cells rather than in CD4^+^ T were present specifically in SS (65). The total percentage of CD27^−^CD57^−/+^CD45RA^+^CD8^+^T lymphocytes with a high level of perforin and killing activity in the peripheral blood of SS patients was lower than that of healthy subjects (15, 66). In contrast, Sudzius et al. proposed that the absolute number of effector CD27^−^CD8^+^ T cells was increased (67).

Of note, T lymphocytes in the blood were negatively correlated with CD45^+^ immune cells infiltrating the glands and the frequency of activated HLA-DR^+^ cells in target tissues, indicating that the reduction in peripheral effector T cells is probably due to their accumulation in the glands. Furthermore, effector memory CD8^high^CD27^+^CD57^−^ T cells with reduced cytotoxicity were significantly reduced in the peripheral blood of SS patients, which was shown to be due to increased spontaneous apoptosis or a sedentary response in the tissues (15, 68). We hypothesize, these cells migrate to local inflammatory sites during progressive disease and are responsible for destroying pathogens. According to some studies, CD27^−^CD57^+^CD8^+^T cells, a subset of CTLs, are potentially highly cytotoxic, but they are destined to migrate to non-lymphoid tissues without further circulation, which is consistent with the above hypothesis (68). At present, a number of studies have shown that mature effector CD8^+^ T cells no longer participate in peripheral circulation throughout disease progression. In advanced SS, CD8^+^ T cells are likely to reside in glandular tissues to exert immune effects. Data show that after 10 weeks of immunization, CD8^+^ T lymphocytes migrate and re-infiltrate with the accumulation of large amounts of the chemokine CCL22 and macrophages in the spleens or salivary glands of SS mice (69).

### Distribution of CTLs in the Major Salivary Glands

**Figure 1:**
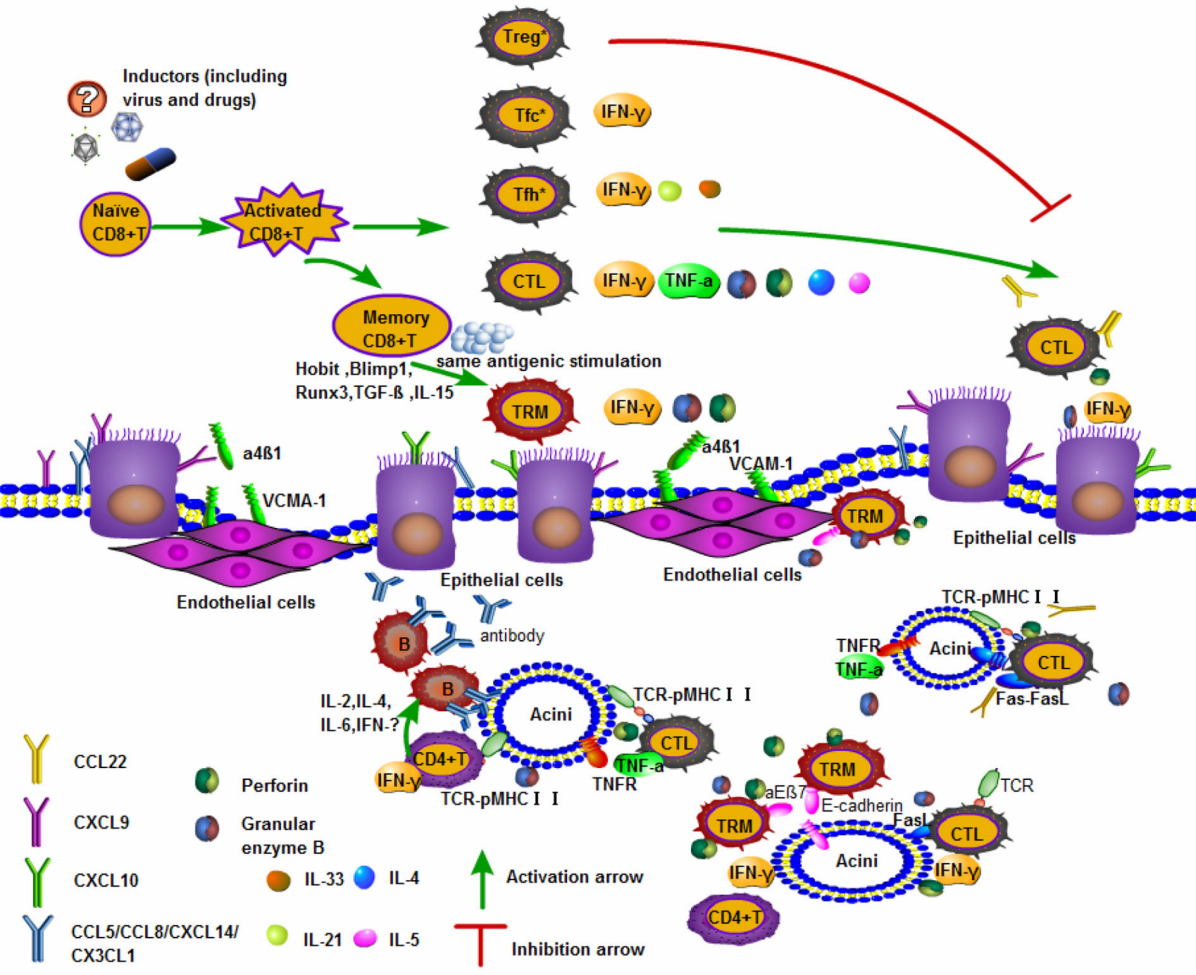
Activated CD8^+^T cells are involved in the mechanism of salivary gland injury. The upper part of the panel shows naïve CD8^+^ T differentiated into subgroups. CTLs and memory CD8^+^ T cells play important roles in the organization. The lower part of the panel highlights the tissues damage caused by activated CD8^+^ T entering the salivary glands. Pathological examination of apoptotic acinar cells in the salivary glands and lacrimal glands of patients with SS revealed the accumulation of CD8^+^ T cells and fewer CD4^+^ T cells expressing integrin *α*E*β*7 (CD103) (12). A kind of cytotoxic memory T cell called CD8^+^ T_RM_ cells and CTLs producing GrB/perforin can induce acinar cells death (53, 72). CTLs recognize pMHCI presented by the acinar cells, then specific T cell receptor (TCR) and co-receptors to contact parts, promoting the secretion of such as IFN-*γ*, TNF-α, GrB/perforin. In addition, CTLs can express FasL or secrete TNF-α, which bind to Fas and TNF receptors (TNFR) on the surface of acinar cells respectively and induce apoptosis mediated by caspase signaling pathways (73, 74). There are also a large number of inflammatory cells such as CD4^+^T cells and B cells in the glandular tissue, which cooperate with CD8^+^T cells to play an immune effect and cause tissues damage. Treg *: CXCR5^+^Foxp3^+/−^CD8^+^Treg; Tfc *: CXCR5^+^PD-1^int-^CD8^+^Tfc; Tfh *: CXCR5^+^PD-1^high^CD8^+^Tfh.

The major salivary glands include the parotid, submandibular, and sublingual glands. Previous reports have shown an increase in IFN-*γ*, CXCL9 and CXCL10 in the salivary gland epithelial cells of SS patients, as well as constant infiltration of CTLs in inflammatory lesions in salivary glands in the female NOD murine model, which is representative of SS (35) IFN-*γ* derived from CTLs can alter tight junction integrity and function in parotid epithelial cells, leading to cell death (70, 71) (**Figure 1**).

Recently, it was found that in aged Lgals1^−/−^ mice (similar to SS, with decreased salivary and increased levels of anti-dsDNA, anti-nuclear and anti-SSA/Ro autoantibodies), the frequency of CD8^+^ T cells in submandibular glands was significantly increased. However, there was no significant difference in the frequency of CD4^+^ T and B220^+^ B cells. In particular, CD8^+^IFN-*γ* ^+^ T and CD8^+^PD-1^+^ T cell infiltration was higher. Consistent with the higher expression of CXCL9 and CXCL10 in submandibular glands, CD8^+^CXCR3^+^ T cells’ infiltration increased as well, compared with WT mice (36). It suggested that CTLs could be actively recruited into this target organ. Kong et al. reported the expression of Fas and FasL in the duct lumen of salivary glands in SS patients (73). This finding suggested the occurrence of apoptosis and progressive damage to salivary glands through CTL-mediated cytotoxicity or the Fas-FasL pathway.

Furthermore, it has been well demonstrated that major salivary gland vascular endothelial cells express the cellular adhesion molecule VCAM-1, which is induced by systemic inflammation and binds with integrin *α*4*β*1 (the ligand of VCAM-1) (75). VCAM-1 promoted the migration of CTLs to exert immune effects in salivary glands, indicating that inflammation enhances T cell recruitment to the salivary glands. The salivary glands, particularly the parotid and submandibular glands, express many chemokines, such as CCL5, CXCL9, CCL28, CXCL14 and CX3CL1, while CD8^+^ T lymphocytes express chemokine receptors, such as CXCR4 and CXCR6, after stimulation (76) (**Figure 1**).

Therefore, we hypothesized that the destruction of salivary glands in advanced SS could be mediated by integrins, chemokines, and activated CD8^+^ T lymphocytes under sustained inflammatory stimulation, following the secretion of inflammatory factors.

### Distribution of CTLs in the Lacrimal Glands

A large number of CTLs expressing IFN-*γ* surround apoptotic acinar epithelial cells of the lacrimal gland in SS patients (11). Moreover, the increased levels of CXCL9 and CXCL10 in the tears of SS patients are consistent with CTL-mediated IFN-*γ* induction (37). Subsequently, researchers demonstrated the presence of proliferating and activated CD11a^+^CD69^+^CD8^+^ T cells, which produced inflammatory molecules in the lacrimal glands of a NOD mouse model of SS (11). In particular, apoptosis mediated by the Fas-FasL interaction was observed in lacrimal acinar cells surrounded by CD107a^+^CD8^+^T cells, while FasL was only detected in SS patients (74, 77). Since CTLs expressing CD107a and GrB can kill the target cell directly, it has been suggested that the death of lacrimal epithelial cells could be independently mediated by CTLs (11). This finding is sufficient to show that CTLs are distributed in the lacrimal glands and play a pathogenic role in SS.

## CD8^+^ T Lymphocytes as Memory T Cells

Memory CD8^+^ T lymphocytes differentiate from naïve CD8^+^ T cells and express CD45RO rather than CD45RA after antigen activation. Initially, memory CD8^+^ T lymphocytes predominantly circulate in the peripheral system and are divided into two classic subgroups: central memory (T_CM_) and effector memory (T_EM_) cells (78, 79). T_CM_ cells are similar to naïve CD8^+^ T cells, express CCR5 and are present in secondary lymphoid organs, while T_EM_ cells express CD45RA and exhibit cytotoxicity similar to that of CTLs in peripheral tissues (54, 80) (**Table 1**). Recently, a new subset of memory CD8^+^ T cells called tissue-resident memory CD8^+^ T lymphocytes (T_RM_ cells) has been widely studied and expresses four classic surface markers: CD44, CD49a, CD69 and CD103 (53). This group is actively involved in disease progression, resides in peripheral tissues and is different from circulating memory CD8^+^ T cells, which will be investigated in detail.

CD8^+^ T_RM_ cells circulate continuously in peripheral tissues and play a leading role in fighting peripheral infections, sometimes mediating stronger protection than any other memory T cells (81). CD44 could not be used to distinguish T_RM_ cells from other CD8^+^T cells, but it could distinguish naïve, effector and memory cells according to the degree of expression. CD44 can bind to vascular endothelial cells to promote peripheral tissue cell migration during inflammation. CD49a is involved in T_RM_ cells production of IFN-*γ*, GrB, and perforin, and it also interferes with T_RM_ cell apoptosis in combination with collagenase type IV (82). In addition to CD8^+^ T_RM_ cells, CD69 is also expressed in other killer cells, such as NKs, and antagonizes SIPR1 to promote tissue retention (83).

Furthermore, there is increasing evidence of T_RM_ cells in autoimmune diseases and inflammatory responses. Recent pathological biopsies of patients with type 1 diabetes mellitus (T1D) showed that there were CD8^+^ T_RM_ cells in the pathological islets, which were prone to producing inflammatory cytokines, including IFN-*γ*, IL-18 and IL-22 (84). Considering that CD103 on the surface of CD8^+^ T_RM_ cells can bind E-cadherin on epithelial cells, CD8^+^ T_RM_ cells are mainly present in mucosal tissues such as the respiratory tract, digestive or urogenital tract, secretory glands and skin (12, 53). In particular, the labial and submandibular glands, as exocrine glands, contain a large number of IFN-*γ*- and GrB-producing CD8^+^ T_RM_ cells (11, 75). In murine SS, the salivary glands are typically damaged, which could be explained by the infiltration of pathogenic CD103^+^CD8^+^T_RM_ cells in glandular tissues (55). It is worth noting that CD103^+^CD8^+^ T cells expressing IFN-*γ* appear to be controversial since they are regulatory cells.

### Distribution of T_RM_ Cells in the Minor Salivary Glands

Minor salivary glands (MSGs) are widely distributed in the lips, tongue, and palate. Although previous reports on the pathogenesis of SS indicated that CD4^+^ T cells were dominant in T lymphocyte-infiltrated lesions, in the labial salivary glands of SS patients, it was noted that there was a group of CD8^+^ T lymphocytes that outnumbered CD4^+^ T cells (55, 85). CD8^+^ T cells with a T_RM_ phenotype secrete high levels of IFN-*γ* and are mainly localized near the duct epithelial cells and acinar cells of the MSGs (55). This finding could be logically explained by CD103 binding to E-cadherin on MSGs epithelial cells (12) (**Figure 1**).

### Distribution of T_RM_ Cells in the Major Salivary Glands

The striking thing is that the majority of infiltrating T lymphocytes in the submandibular glands of p40^−/−^CD25^−/−^ mice, which recapitulate the main characteristics of human SS, are CD8^+^ T lymphocytes. They exhibit a T_RM_ phenotype instead of CD4^+^ T cells, accompanied by significantly increased IFN-*γ* (55). CD8^+^ T cells are also a source of IFN-*γ* and cause direct destruction of glandular tissues. IFN-*γ* is closely connected to the death of parotid epithelial cells in the salivary glands (70, 71) IFN-*γ* significantly recruits the chemokines CXCL9 and CXCL10 on the surface of submandibular epithelial cells, then immune cells home to lesions, and exacerbate the progression of SS (35, 86) (**Figure 1**).

As CXCR3 expression is increased in T cells in draining lymph nodes, IFN-*γ* may promote CD8^+^T cell migration from draining lymph nodes to submandibular glands and exert effects in tissues, especially in the case of consistent inflammation and infection (55, 86). Therefore, the positive feedback pathway induced through the CD8^+^T-IFN-*γ*-CXCL9/10-CXCR3 axis further exacerbates tissue damage.

Moreover, researchers discovered that acinar atrophy, ductal injury and fibrosis were completely eliminated by knocking out CD8a in p40^−/−^CD25^−/−^ mice, while CD4 knockout provided only mild relief. Moreover, only depletion of CD8a but not CD4 alleviated the lymphocyte lesions in the submandibular glands and restored salivary secretion (55). Tissue damage occurred despite inhibition of the germinal center responses and antibody production in p40^−/v^CD25^v/−^CD4^−/−^ or IFN-*γ* ^−/−^ mice (55). In summary, CD8^+^T lymphocytes, especially CD8^+^ T_RM_ cells, lead to salivary gland tissue damage in mice and may play a role that cannot be ignored in the occurrence and development of SS.

## CD8^+^ T Lymphocytes as Regulatory T Cells

Regulatory CD8^+^ T cells (CD8^+^ Treg cells) are considered to be essential regulators and should be discussed. CD8^+^ Treg cells prevent the progression of autoimmune diseases by secreting a variety of cytokines to inhibit lymphocyte function (87–90). CD8^+^ Treg cells are mostly characterized by CD45RO expression and lack CD28, CD62L or CD122 expression (91, 92). CD8^+^ Treg cells can be divided into natural and induced subgroups according to their origins. Natural CD8^+^ Treg cells are generated and mature in the thymus and express human leukocyte antigen-G (HLA-G) or CD122 and CD28. The inhibitory effects of these cells are mediated by soluble factors such as HLA-G or IL-10 (93, 94). In contrast, induced Treg cells (including effector and memory CD8^+^ Treg cells) are derived from peripheral naïve T lymphocytes, which are stimulated by antigens and exert functions through cell–cell contact or the release of soluble factors (95, 96).

There is no real consensus on the phenotypic and molecular characteristics of regulatory CD8^+^ T cells, which appear to make up a heterogeneous group expressing Foxp3 (26). The phenotypic expression of CD8^+^ Treg cells, including CD122, CD28, CD45RC, CD103, and PD-1, is mainly associated with the differentiation status of CD8^+^ T lymphocytes among central or effector memory cells (97, 98). The inhibitory ability of induced CD8^+^ Treg cells is much stronger than that of naïve CD8^+^ Treg cells (99). Effector CD8^+^ Treg cells are present in the blood and secondary lymphoid organs, while memory CD8^+^ Treg cells are mainly distributed in peripheral tissues. A subset of induced Treg cells expressing CD8^+^CD28^−^ can inhibit the production of high-affinity antibodies and autoantibodies to regulate humoral responses (40, 100). A reduction in the number of CD8^+^CD28^−^ T lymphocytes with inhibitory effects has been described in animal models of autoimmune diabetes and experimental autoimmune encephalomyelitis (EAE) (100–103). The subset CD8^+^CD28^−^Foxp3^+^ T lymphocytes, which are similar to classic CD4^hi^CD25^+^Treg cells, inhibit the development of autoimmune diseases. Previously, it has been shown that CD8^+^CD28^−^Foxp3^+^T downregulate the expression of co-stimulatory molecules in DCs, reducing the efficiency of antigen presentation (104–106), regulating the activity of IDO^+^ plasmacytoid dendritic cells (pDCs), and mediating immune tolerance (104). In addition, CD8^+^ Treg cells could eliminate CD4^+^ Tfh cells by IL-21 and enhance perforin and IL-15 (27).

In addition, this subset includes a specific population called antibody-suppressor (CD8^+^ T_Ab-supp_) cells that produce IFN-*γ*, which directly kills or inhibits IgG and IL-4 secretion by allogeneic B and CD4^+^ T cells (28) (**Table 1**). Reinforcing the notion that the key mediators of CD8^+^ Tregs inhibitory activity are IFN-*γ* and TGF-*β*, IFN-*γ* also participates in the pathogenicity of CTLs (107–110). An advantage is that CD8^+^ Treg cells’ activation is stimulated by almost all cell types, while CD4^+^ Tregs’ activation occurs only through cell expressing MHC-II molecules. CD8^+^ Tregs are useful in organ transplantation or graft-*versus*-host disease (GVHD) and are critical in autoimmune diseases (98, 111, 112).

### Distribution of CD8^+^Tregs in the Blood

A recent study found that circulating regulatory CD8^+^CD28^−^ T cells were negatively correlated with systemic disease activity in patients with SS (95, 113). Sudzius et al. demonstrated that CD8^+^Foxp3^+^ Treg cells showed a decreasing trend in the peripheral blood of pSS patients, even though it was not significantly different (67). Compared with that of healthy controls, the percentage of CD8^+^Foxp3^+^ Treg cells in the peripheral blood of pSS patients without clinical disease activity but with serological activity was significantly reduced, while there were no obvious changes in the frequency of CD4^+^ Treg cells (114). However, with the progression of the disease, advanced SS tissue lesions are exacerbated, and increased Treg cells are detected in the peripheral blood (115). The proportion of CD8^+^CD28^-^T cells in the peripheral blood of SS patients was significantly higher than that of healthy controls, as the level of soluble CD28 was also increased (113, 116). The migration of pathogenic or regulatory CD8^+^ T lymphocytes between the periphery and local disease-affected tissues is thought to occur, as CTLs have been detected in lacrimal and salivary glands (117).

### Distribution of CD8^+^ Tregs at the Ocular Surface

Previous studies demonstrated that the number of infiltrating CD4^+^ Treg cells in the MSGs of SS patients was positively correlated with the gland biopsy lesion score but negatively correlated with the number of cells in the peripheral blood. Therefore, it can be posited that CD8^+^ Tregs exhibit opposing regulation in the peripheral blood and affected tissues, even though this regulation is reversible. Furuzawa-Carballeda et al. showed that IL-10^+^CD8^+^ Tregs migrated to lacrimal and salivary glands and appeared to directly contact target cells to mediate immune regulation (114). In addition to salivary glands and lacrimal glands, the ocular surface is another key site of inflammatory damage in SS. However, other researchers found that instead of causing disease, CD8^+^ T lymphocytes regulate the ocular surface in desiccating stress (DS)-induced SS mice, and so further research is needed. Although natural regulatory CD122^+^CD8^+^ T cells exist in the ocular surface, they seem to be independent of the development of SS (13, 73, 118). Zhang X demonstrated for the first time that CD103^+^CD8^+^ Tregs could significantly reduce Th17-mediated corneal barrier dysfunction in in a murine model of SS by inhibiting DCs activation (13). CD8^+^ T lymphocytes’ depletion increased DCs’ accumulation and activation and promoted DCs’ migration from the ocular surface to cervical lymph nodes (13). While we previously discussed CD103^+^CD8^+^ T cells with pathogenic effects on salivary glands, whether pathogenicity or regulation is related to the presence of these cells in different tissues or to the stage of SS development remains to be further clarified.

Collectively, three kinds of CD8^+^ T lymphocytes have been summarized in this section (**Table 1**). We discussed the different CD8^+^ T subsets that contribute to SS in depth. CD8^+^ T lymphocyte subsets are widely distributed in the peripheral blood and tissues with complex classifications and diverse phenotypes. However, subsets of the same phenotype may exhibit pathogenicity or regulation through different distributions in SS patients. Therefore, because CD8^+^ T lymphocytes have not been extensively reported in detail in SS, we need to further explore the role and influence of CD8^+^ T lymphocytes.

## Targeting CD8^+^ T Lymphocytes: A Promising Treatment for SS

At present, it has been reported in the literature that the pathogenesis of Sjögren’s syndrome is probably related to the up-regulation of type I interferon. Consistent with this, SIGLEC1 (known as sialoadhesin and CD169, responsive protein of IFN-α) on mononuclear cells of SS patients was also significantly up-regulated, which has been proven to be related to the disease activity (119). Besides, B-cell aggregating in ectopic GCs is also pathogenic, producing antibodies and forming immune complexes with autoantigens. It can be deposited in tissues, promoting inflammation (120). However, the pathogenesis of SS is diverse without a complete understanding of the underlying pathogenic factors, and there is currently no specific cure. SS is characterized by systemic dryness, especially dry mouth and dry eyes, which are also observed in SICCA syndrome (1, 4). Therefore, it is difficult to diagnose SS through clinical symptoms, which urges us to look for characteristic indicators such as changes in autoantibodies, cytokines, effector molecules or immune cell subsets for diagnosis. The subsets of CD8^+^ T lymphocytes in SS are a controversial issue because the retention of CD8^+^ T cells in tissues contributes to both pathogenicity and regulation during disease development.

Given the key role of CD8^+^ T lymphocytes in the pathogenesis of SS, CD8^+^ T cell-targeted therapy has great promise. Unfortunately, no satisfactory results have been achieved. However, treatment of the disease with damaged glandular cells may be effective. For example, the proteasome inhibitor lactacystin suppresses the formation of immunoproteasomes in human-SG (HSG) cells by binding to the N-terminal of the *β*1i subunit (121, 122). IFN-*γ* is overexpressed during the progression of SS and up-regulates the immunoproteasome in SG cells, promoting the expression of MHC-I-associated peptides (123). MHC class I may be recognized and targeted by autoreactive CD8^+^T cells and then destroy SG cells in SS patients. Hence, the use of proteasome inhibitors to inhibit the immunoproteasome or targeting peptide epitopes on HSG cells is a promising strategy for the treatment of SS. Moreover, the direct use of MHC class I and self-peptide complexes can selectively induce autoreactive CD8^+^T cells apoptosis (124). Furthermore, blocking effector molecules associated with CD8^+^T cells might also be used to reduce the cytotoxicity and apoptosis of glandular epithelial cells or acinar cells.

On the other hand, improving SS may occur through targeting CD8^+^ T lymphocytes directly. Barr et al. demonstrated that NOD mice had lacrimal inflammation and cytotoxic CD8^+^ T cell infiltration, and the lack of CD8^+^ T cells reduced disease severity. In fact, cyclosporine inhibits the proliferation of CTLs *in vitro* (125). Douglas A. treated MRL/lpr mice with cyclosporine at the early stage and found that cyclosporine was effective in controlling autoimmune diseases, especially in alleviating lacrimal glands and intraocular inflammatory lesions (126). Cytotoxic T cell antigen 4 (CTLA-4), which can block the binding of B7 to CD28, leading to immune tolerance. CTLA-4Ig is a fusion protein composed of CTLA-4 extracellular functional genes and Fc segments of IgG1, which can destroy T cell activation. This may be a potential treatment for inhibiting the progression of pSS through suppressing CD8^+^ T activated in tissues. Meiners PM et al. found that abatacept treatment was effective, safe, and well tolerated in early pSS patients, because it resulted in a significant reduction level of ESSDAI and EULAR Sjögren’s syndrome Patient Reported Index (ESSPRI) (127). However, in the single-center abatacept Sjögren Active Patients phase III (ASAP-III) study, there was no difference between pSS patients treated with abatacept and those treated with placebo measured by ESSDAI or ESSPRI (128). Baer AN et al. also suggested that abatacept did not produce significant clinical efficacy compared with placebo in patients with moderate or severe pSS. Limitations in this study may be the heterogeneity of patients and missing CD8^+^ T cell population detection. While they detected improvements in some disease-relevant biomarkers and pathogenic cell populations such as CD4^+^T, it is difficult to determine whether it is effective against CD8^+^ T. Therefore, further studies are needed to assess the effect of abatacept on specific disease-relevant CD8^+^ T cells changes of pSS (129). In addition, the number of CD8^+^ Treg cells was negatively correlated with disease activity, regulating SS development (95, 113, 115). Thus, CTL depletion and powerful CD8^+^Tregs amplification may be achieved in early SS when certain drugs like abatacept and cytokines are applied.

Currently, new targets under investigation, including several approaches discussed in this review, which may intervene in the involvement of CD8^+^ T lymphocytes in SS, are shown in **Table 2**. Advances in our understanding of the pathogenic or regulatory mechanism of activated CD8^+^ T subsets are expected to provide effective treatments for SS patients in the future.

**Table 2:** Potential therapies for SS by targeting CD8^+^ T cells.

Type of therapy	Target of therapy	References
Abatacept (CTLA-4 Ig)	Blocking the binding of B7 to CD28, destroying T cells’ activation	(127, 129)
Galectin-1 (Gal1)	Reduction of CD8^+^IFN-*γ* ^+^T or CD8^+^PD-1^+^ T cells infiltration of salivary glands	(36)
Immunoproteasome*β*1i inhibitor	Immunoproteasome formation in SGs	(121, 122)
MHC class I and the self-peptide complexes	Autoreactive CD8^+^ T cell response	(124)
Anti-pDC mAb	Type I IFN secretion	(130)
Anti-CD40 mAb	Downregulation of the molecule c-FLIP to promote Fas-mediated SGEC apoptosis, inhibition of B cells	(131–134)
CD8^+^T cell depletion (cyclosporine)	Apoptosis or death of lacrimal acinar cells	(11, 125)
Rapamycin	Expansion of human CD8^+^ Treg cells *in vitro* for clinical application	(91, 135)
IL-15	Expansion of CD8^+^ Treg cells *in vivo* or *in vitro*	(136)
IL-34	Induction of regulatory macrophages to regulate the CD8^+^ Treg/Teff balance	(137)
Demethylation of Foxp1	Mediating Foxp3 DNA binding and increasing CD8^+^Treg cells suppressive functions	(138)

## Conclusion

The exocrine glands of SS patients have different degrees of inflammatory cell infiltration or ectopic germinal centers. In particular, activated CD8^+^ T lymphocytes in the tissues in SS are characterized as cytotoxic, promoting apoptosis or even inhibiting the disease, and these findings have been shown in current studies and support the idea that these cells are actively involved in the development of SS. The targets of this disease, salivary gland and lacrimal gland epithelial cells, maintain cross-talk with activated CD8^+^ T subsets. Environmental triggers occur through heredity, viral invasion and estrogen imbalance, leading to the activation of specific CD8^+^T cell subsets, especially T_RM_ cells that reside in tissues and exhibit high cytotoxicity, which mediate glandular cell apoptosis through the Fas death pathway or releases toxic particles to target cell destruction (81, 85). Whether CD8^+^ T lymphocytes can induce or exacerbate the destruction of lacrimal and salivary glands remains to be further explored. Furthermore, the evidence for SS discussed in this review suggests that targeting CD8^+^ T cells may be a complementary strategy for disease immunotherapy. In summary, we explored evidence for the participation of CD8^+^ T lymphocyte subsets in the pathogenesis or regulation of SS with the hope of gaining insights to enhance CD8^+^T cell-targeted therapies.

## Author Contributions

HZ drafted the manuscript. JY and JT discussed and revised the manuscript. SW designed the study and revised the manuscript. All authors contributed to the article and approved the submitted version.

## Funding

This work was supported by the National Natural Science Foundation of China (Grant Nos. 81971542, 81771759) and Jiangsu Province’s Key Medical Talents Program (Grant No. ZDRCB2016018).

## Conflict of Interest

The authors declare that the research was conducted in the absence of any commercial or financial relationships that could be construed as a potential conflict of interest.
